# A Quality Assurance Audit of an Orthoptic-Led Virtual Neuro-Ophthalmology Clinic

**DOI:** 10.22599/bioj.289

**Published:** 2023-03-10

**Authors:** Joe Smith, Nicola Hirst, Ali Yagan

**Affiliations:** 1 Advanced Orthoptist, GB; 2 Principal Orthoptist, GB; 3 Consultant Ophthalmologist – Neuro-Ophthalmology and Adult Strabismus, GB

**Keywords:** neuro-ophthalmology, virtual clinics, orthoptic-led, shared-care service models, AHP-led

## Abstract

**Introduction:**

Neuro-ophthalmology is experiencing increasing demand. There is a lack of literature relating to how we deal with the capacity and demand issues, and the use of virtual clinics. Virtual clinics are well-recognised in other subspecialties to help deal with capacity and demand issues, with reports of non-medical-led clinics, and are supported by the National Health Services Long Term Plan and People Plan. We present a quality assurance audit of our orthoptic-led neuro-ophthalmology virtual clinic.

**Methods:**

The virtual clinic was developed in October 2014 to help deal with capacity and demand issues, however, only since 2019 have orthoptists been trained to virtually review. This paper provides a summary of a quality assurance audit that was completed to determine concurrence between orthoptic and consultant virtual reviews.

**Results:**

From January to August 2021, all patients attending the virtual clinic were reviewed by a trained orthoptist and then audited for concordance by a consultant neuro-ophthalmologist; 163 patients were identified. There was 100% agreement between orthoptic reviewers and consultants regarding visual function status and optical coherence tomography interpretation. Of the patients reviewed, 100% of patients were satisfied and 96% of patients would be happy to be seen in the virtual clinic again.

**Conclusions:**

Current demands on the neuro-ophthalmology speciality mean that we need to explore new ways of working. Our outcomes demonstrate a high level of agreement between orthoptists and ophthalmologists, and excellent patient satisfaction results. We hope this highlights where orthoptists can play an invaluable role in the care of neuro-ophthalmic patients and help deal with capacity and demand issues.

## Introduction

Along with other ophthalmic subspecialties, neuro-ophthalmology is experiencing increasing demand in more recent times, which has been exacerbated by the COVID-19 pandemic. Before this, Ophthalmology was already recognised as the busiest outpatient speciality, with an expected further 30–40% increase in demand over the next 20 years (NHS Digital 2019; The Royal College of Ophthalmologist 2018). Virtual clinics are a well-recognised model of service delivery in other subspecialties such as medical retina (Lee *et al*. 2018; Kortuem et al. 2018; El-Khayat et al. 2018) and glaucoma (Gunn et al. 2018; Nikita et al. 2021; Koklanis and Thorburn 2021; Simons et al. 2021). There is also reported use of virtual clinics for the identification and referral of ocular melanocytic lesions (Karthikeyan et al. 2021). Virtual interactions have the potential to free up clinician time and appointment slots by reducing the time and space required for patient interactions and reducing did not attend rates (NHS England 2016). Virtual clinics and other forms of remote consultation within neuro-ophthalmology are not well documented in the literature before the COVID-19 pandemic. More recently reports of the use of telemedicine are emerging, however, many of these reports outline the options for use within the neuro-ophthalmology setting rather than comparing the validity of a remote model compared to a face-to-face model (Grossman et al. 2020; Ko and Busis 2020; Lai et al. 2020). Nevertheless, these reports are valuable in providing solutions for transforming the way that outpatient consultations are delivered, to address the imbalance between capacity and demand. A survey by Moss et al. (2020) reported an increase in the remote interpretation of tests in a neuro-ophthalmology setting from 27.6% pre-COVID to 32.2% during COVID.

Delivering neuro-ophthalmology consultations remotely is difficult given the need for access to the physical examination and results from diagnostic testing to differentiate benign conditions from sight- and life-threatening neurologic emergencies (Grossman et al. 2020). This may be a reason why face-to-face interactions have taken precedence over virtual consultations in the past. Lai et al. (2020) suggested that the combination of various modalities may improve efficacy; initial phone triage and an in-person appointment for assessment followed by possible video consultation. The availability of ancillary testing will determine the ability to follow a patient remotely for optic nerve pathology, such as idiopathic intracranial hypertension, which in addition to history, requires a fundus view, a visual field, and optical coherence tomography (OCT) to determine clinical stability. The effective use of a virtual clinic model for patients referred with suspected optic nerve swelling has been reported by Jefferis et al. (2021). One hundred and thirty-three patients were virtually reviewed, retrospectively, by two consultant neuro-ophthalmologists who were blinded to the outcome of the face-to-face clinic. From the simulated virtual clinics, they found six out of one hundred and thirty-three had papilloedema and identified these as needing a face-to-face assessment. One hundred and twenty (90%) were discharged from face-to-face clinics and two ophthalmologists chose to discharge 114 and 99 respectively from the simulated virtual clinics. Only one patient would have been missed with serious pathology who had normal optic discs but reported diplopia at the face-to-face consultation (Jefferis et al 2021). Unrelated pathologies may be missed by solely reviewing the image without further information which supports the combination of using various virtual modalities as suggested by Lai et al. (2020), for example, including clinical questions as part of the virtual consultation to gather more information. Such reports support the development of neuro-ophthalmology virtual clinics and demonstrate their safety and effectiveness.

The use of the non-medical workforce to help address challenges with capacity and demand is supported by the National Health Service (NHS) Long Term Plan and the NHS People Plan (NHS England 2019; NHS England 2020). To deal with the increasing demand for ophthalmic services, The Royal College of Ophthalmologists also recognised that new models of care must encompass maximum use of consultant time and expertise and enhance the multidisciplinary team with appropriate training and upskilling (The Royal College of Ophthalmologists 2017). Investing time and training in the non-medical workforce will help ease pressure on already stretched ophthalmic services. In the United Kingdom (UK), the Royal College of Ophthalmologists has worked with the British and Irish Orthoptic Society and other professional bodies to support the development of clinical competency frameworks to support the professional development of the multi-disciplinary eye health team. The Ophthalmic Common Clinical Competency Framework (Health Education England 2016) sets out standards for a systematic patient-centred approach to multi-disciplinary education and training, ensuring standardised and recognised competencies.

The reports and plans discussed above highlight the need to change the way we deliver services and support the use of the non-medical workforce as a solution to capacity and demand issues. In this article, we present an audit of our orthoptic-led neuro-ophthalmology virtual clinic. We believe this to be the first report of a UK non-medical-led virtual clinic in neuro-ophthalmology.

## Methods

### Virtual clinic

The Neuro-Ophthalmology and Orthoptic department at Manchester Royal Eye Hospital developed the virtual neuro-ophthalmology service in October 2014 to monitor and review patients with non-complex and stable neuro-ophthalmic conditions. The virtual clinic was established due to increasing demand for the neuro-ophthalmology service and aimed to streamline the patient assessment and reduce the number of patients waiting to be seen in a consultant-led clinic. Since the origin of the clinic, many service developments have been made based on regular clinical audits and increasing clinical demands.

### Clinical model

At the time when this audit was being completed the virtual neuro-ophthalmology clinic ran twice weekly, with eight patients per session. Four of the patients attending were new patients with suspected optic nerve head swelling and were reviewed by a neuro-ophthalmology clinical fellow and the remaining 12 patients were follow-up patients who had varying diagnoses and were deemed as suitable for review by the orthoptic reviewers. Since this audit, the clinics have been remodelled and now run as four separate clinics, twice weekly, with four patients per clinic. The clinics are as follows 1) Idiopathic Intracranial Hypertension (IIH); 2) Tumour Monitoring; 3) Suspected Swollen Optic Nerves; and 4) General. The orthoptic reviewers review all patients attending the IIH and tumour monitoring clinic, and the neuro-ophthalmology fellows or consultants review all patients attending the suspected swollen optic nerves and general clinic. The general clinic includes patients with optic neuropathies and visual function abnormalities that do not fit the inclusion criteria for the other virtual clinics. Patients are migrated to the virtual clinics if deemed non-complex and not requiring face-to-face review by the medical team (e.g. consultant).

### Orthoptic reviewers

In 2019, a business case was drafted, due to the increasing demand for the neuro-ophthalmic service, which proposed the development of new posts in which orthoptists would be upskilled to virtually review more stable patients who do not require face-to-face appointments. The business case included a full options appraisal (e.g. do nothing, do the minimum – such as develop waiting list initiatives, or develop these new posts) and risk assessment (e.g. quality, safety, and operational effectiveness). The business case was successful, with two orthoptists being appointed and trained to perform the virtual reviews. Both orthoptists had experience working with neuro-ophthalmic patients. One had previously completed an MSc module in neuro-ophthalmology (ORTH413: Specialist and Extended Roles in Neuro-Orthoptics and Neuro-Ophthalmology at the University of Liverpool) and the other was completing an MSc Advanced Clinical Practitioner course with optional neuro-ophthalmology modules (ORTH410 – Neurology for Advanced Clinical Practice and ORTH413 Neuro-ophthalmology for Advanced Clinical Practice at the University of Liverpool). Before independently reviewing the notes of patients that attended the virtual clinics, both orthoptists reviewed cases and attended feedback sessions in which they discussed the cases with a consultant ophthalmologist and received feedback. The supervising ophthalmologist would also use these sessions to teach the orthoptist about neuro-ophthalmic conditions, discussing presenting signs and symptoms, and the investigations required for diagnosis and management, with comprehensive discussions. These sessions were weekly and took place for over a year. Both orthoptists also worked in outpatient clinics and demonstrated competency in identifying visual function changes and posterior segment pathologies. Specific training for assessing optic disc pathologies using optical coherence tomography was undertaken with an external education provider. Finally, before deeming the orthoptic reviewers competent, a competency package was also completed that evidenced the ability to perform virtual reviews independently and ensure concerns were brought to attention before autonomy was achieved. A decision document (Table 1) was also produced to assist the orthoptic reviewers in making decisions and when cases needed to be brought to the attention of the ophthalmologist.

**Table 1:** Neuro-ophthalmology virtual review decision document.


CONDITION	FINDINGS	FOLLOW-UP

***Tumour monitoring***	Stable visual functions and OCT findings ± no new symptoms ± stable MRI	12 months

	Worsening of visual functions ± new symptoms	Inform consultant or clinical fellow Arrange urgent F2F review Letter to relevant speciality ± arrange MRI

***Idiopathic intracranial hypertension***	Stable visual functions and OCT findings (no papilloedema to grade 1) ± no new symptoms ± tolerating medication	6 months Counsel regarding weight loss Counsel about pregnancy if on medications To consider stopping medications

	Worsening of visual functions ± new symptoms ± not tolerating medication	Inform consultant or clinical fellow Arrange urgent F2F review or phone consultation

***Stroke and unexplained visual field defects***	Stable visual functions and OCT findings ± no new symptoms ± stable/normal MRI	Discharge

	Worsening of visual functions ± new symptoms	Inform consultant or clinical fellow Arrange urgent F2F review or phone consultation

***Optic neuropathies***	Improving/stable visual functions and OCT findings ± no new symptoms ± MRI confirmed ON or normal in AION ± Stopped steroid or under rheumatology if GCA	Discharge

	Worsening of visual functions ± new symptoms	Inform consultant or clinical fellow Arrange urgent F2F review or phone consultation


### Clinical assessment

**Figure 1:**
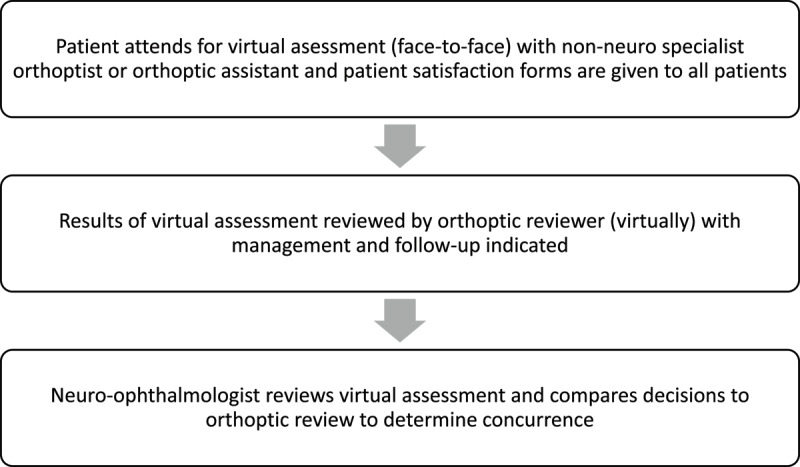
This flowchart details the patient flow through the virtual clinic during the time of the audit. All patients Attended for virtual assessment with the results then reviewed by an orthoptic reviewer with decisions made that were then checked for concurrence by a neuro-opthalmologist.

At the clinical assessment, depending on diagnosis, each patient has a bespoke pro forma completed. Three bespoke pro formas are used to take a clinical history and assess patients (Appendix A). These are 1) IIH; 2) compressive and general aetiologies; and 3) suspected swollen disc. Bespoke pro formas are used to ensure all relevant clinical information is obtained at the initial clinical assessment, including history and diagnostic assessments, as the initial diagnostic assessment is completed by a non-neuro specialist orthoptist or orthoptic assistant. The pro forma is filed into the patient’s notes and given to the reviewer following the clinic. At each visit, all patients have visual acuity assessed using a logMAR acuity chart, colour vision assessed using the Ishihara, pupil reactions, visual fields (30–2 or Goldmann Perimetry in tumour monitoring patients and when previous static perimetry has been deemed unreliable), and optical coherence tomography of the optic disc and macula (TOPCON or Heidelberg). The images from the optic coherence tomography are viewed on a networked desktop. Figure 1 shows the clinic flow during the time of this audit.

### Quality assurance

During the time of this audit, all virtual reviews were checked by a consultant to ensure concurrence. Following the orthoptic reviewers being deemed competent, this is not necessary, however, assurance checks are continually undertaken to ensure no patients come to any clinical harm. Continuous monitoring is achieved by the consultants reviewing several complete virtual review sessions every four months to ensure full agreement with decisions made by the orthoptic reviewers. Reviews are also monitored on a random basis to ensure quality is consistently maintained. All virtual clinics are audited at two yearly cycles based on clinical governance requirements of the Trust. If issues are identified, the clinic is audited earlier and more regularly until assurance is fully assured. All patients attending the virtual clinics are also given a patient satisfaction survey to complete and data is collected and reviewed regularly to help make appropriate changes and improvements to the clinics, if deemed necessary.

## Results

### Audit of service

From January to August 2021, all patients attending the neuro-ophthalmology virtual clinic were reviewed by a trained orthoptist and then reviewed for concordance by a consultant neuro-ophthalmologist; 163 patients were identified. Of these patients, 62 had a diagnosis of IIH, 88 with a compressive aetiology (included tumours, venous thrombosis, and aneurysms), three with optic neuropathies, two with swollen discs with unconfirmed diagnoses, two with crowded discs, one with a tilted disc, one with systemic lupus erythematosus, one with optic nerve head drusen, one with migraine, one with a field defect, and one that had an unremarkable ophthalmic assessment.

### Correlation between orthoptist and consultant

There was 100% agreement between orthoptic reviewers and consultants regarding visual function status and optical coherence tomography interpretation. The level of agreement dropped to 95% (155/163) when it came to follow-up periods. The reason for the drop in agreement was mainly due to the orthoptic reviewer being overly cautious and reviewing patients sooner than deemed clinically necessary by the consultant. For example, the orthoptist would review a patient with a stable pituitary adenoma and visual functions in six months whereas the consultant deemed the patient would be safe to wait 12 months for clinical review. Unfortunately, as orthoptists cannot currently prescribe, there were occasions when the orthoptist was required to bring the patient to a face-to-face consultant clinic so a new medication could be prescribed whereas the consultant could prescribe the medication virtually and then arrange a normal review. This, however, does not cause clinical harm or visual compromise.

### Patient satisfaction

The results of anonymised patient satisfaction questionnaires following virtual clinical assessment are presented, 145/163 (88.9%) completed the questionnaires. A summary of the responses is as follows:

- Do you feel the appointment was quicker than an appointment to see the ophthalmologist face-to-face? Yes 100%
- Were you given the opportunity to ask any questions at the assessment? Yes 100%
- Were you satisfied with the response? Yes 100%
- How would you rate your overall satisfaction with today’s appointment? Very Satisfied 74% and Satisfied 26%
- Would you be happy to be seen in the virtual clinic again? Yes 96%

Thirty-nine patients were randomly selected to contact following virtual review to ensure they were happy with their experience at the clinic and the quality of the letter they received from the orthoptic reviewers. Patients were contacted by phone call and 18 out of 39 patients were available to talk or responded to the voicemail left for them. Responses were as follows:

- Did you receive a letter explaining the findings from the virtual appointment? Yes 94% No 6%
- Was it clear from your letter which clinic your follow-up would be in, face-to-face or virtual clinic? Yes 89% No 11%
- Was it clear from the letter when your follow-up appointment would be? Yes 94% No 6%
- Did you feel satisfied with the amount of information provided in the letter? Yes 100%
- Would you be happy to be booked into the virtual clinic again? Yes 94% No 6%

The negative responses related to the explanation of clinical findings referred to the use of medical terminology in letters that patients did not understand. To overcome this orthoptists have replaced such terms with more patient-friendly descriptions. For example, instead of referring to the OCT of the optic nerve, use of terms such as, the pictures or scans of the back of the eye. It was also clear that several letters did not have the specific clinic (e.g. face-to-face or virtual) and/or timeframe for the follow-up included. This has been improved by including clear instructions for follow-up at the top of letters. Finally, two patients reported that they would not be happy to be seen in the virtual clinic again, and this was because they had questions to ask on the day. This issue has been resolved by including a section for patient questions on the clinical pro formas with an option for the patient to be called with results.

## Discussion

We believe this to be the first report of a UK orthoptic-led neuro-ophthalmology virtual clinic and we hope it highlights where orthoptists can play an invaluable role in the care of selected patients within the neuro-ophthalmology service. There is evidence of this within other ophthalmic subspecialties. El-Khayat et al. (2020) found in a virtual clinic setting, 100% agreement between three ophthalmic photographers and doctors in management decisions in patients with macular oedema secondary to diabetes or retinal vein occlusion during a 300-patient observation period. This resulted in a significant increase in the number of patients seen within clinics. A study by Koklanis and Thorburn (2021) evaluated the safety of an orthoptist-led glaucoma-monitoring clinic and found a substantial agreement of 85.71% between the orthoptists and ophthalmologists for the clinical management decisions of patients diagnosed with glaucoma. Furthermore, a study by Karthikeyan et al. (2021) found 98.6% concordance between optometrists and consultants for the diagnosis and management of patients with ocular melanocytic lesions. These numbers are very similar to the ones presented in our audit and support the safety and effectiveness of orthoptic-led virtual neuro-ophthalmology clinics. It is, however, important to have stringent quality assurance measures in place to ensure continuing safety and effectiveness. We discussed how we ensured this by consultants regularly reviewing patient records and performing quality assurance checks.

Another important aspect of governance to consider, when developing new models of service delivery, is patient and service user input. We audited patient satisfaction and received excellent feedback from our patients with 100% of patients being very satisfied or satisfied with our model of service delivery, and 96% of patients being happy to be booked into the virtual clinic again. Karthikeyan et al. (2021) also reported that 100% of patients were satisfied and 95% would be happy to continue to be monitored by AHPs in their paper. This again supports the development of non-medical-led virtual clinics.

As well as being a safe and effective model of service delivery, our clinic makes use of the non-medical workforce to help address challenges with capacity and demand. As mentioned earlier, this is in line with the NHS Long Term Plan and the NHS People Plan, as well as the Royal College of Ophthalmologists *The Way Forward*, which recommends working in new ways and utilising other health professionals for advancing the scope of practice (NHS England 2019; NHS England 2020; The Royal College of Ophthalmologist 2017). Our model of service delivery gives orthoptists more clinical responsibility which improves job satisfaction and enables continuous professional development. Operational effectiveness is also maintained as patients are reviewed in the same format and there is no decrease in clinical activity. In addition, this is an opportunity for cost-saving, as despite requirements for high banding of orthoptists (our orthoptists are Agenda for Change Band 7 and above), it costs less per session to fund an orthoptic reviewer. This model of service delivery does depend on the competence of orthoptists within the department, but we do present how competency was achieved with our orthoptists in the *orthoptic reviewers* section of this paper. Utilising the non-medical workforce means that ophthalmologists’ time can be utilised more effectively, meaning that higher-risk patients can be reviewed face-to-face in a timelier manner.

Finally, it is important to recognise that this paper is based on a quality assurance audit and only looks at a select number of patients over a short period of time. Future papers should include a greater number of patients over a longer period. Such papers will serve to support the development of further non-medical-led (virtual) clinics within the NHS and beyond, ensuring we can address the increasing challenge of capacity and demand.

## Conclusion

In conclusion, the demands on the neuro-ophthalmology sub-speciality mean that we need to explore new ways of working. Non-medical led (virtual) clinics are well documented in other ophthalmic sub-specialities, and these models of service delivery are crucial to ensuring the safe and effective delivery of care to patients in a timely manner. Our outcomes demonstrate a high level of agreement between orthoptists and ophthalmologists, and excellent patient satisfaction outcomes. This shows that select neuro-ophthalmic patients can safely be monitored by orthoptists in a shared care model with a clear escalation protocol. We hope to have highlighted where orthoptics can play an invaluable role in the care of selected patients within the neuro-ophthalmology service. Future papers should include a greater number of patients over a longer period to demonstrate continuing effectiveness over time.

## Additional Files

The additional files for this article can be found as follows:

10.22599/bioj.289.s1 Appendix A-1. IIH Proforma.

10.22599/bioj.289.s2 Appendix A-2. General and Tumour Monitorting Proforma.

10.22599/bioj.289.s3 Appendix A-3. Swollen Discs Clinic – Questionnaire.
