# Supplementary material for: A Quality Assurance Audit of an Orthoptic-Led Virtual Neuro-Ophthalmology Clinic
Source: Br Ir Orthopt J. 2023 Mar 10;19(1):7–14. doi: 10.22599/bioj.289 (PMC10000316; doi:10.22599/bioj.289)
Supplement: Appendix A-1. — IIH Proforma. [file bioj-19-1-289-s1.pdf]

IIH PROFORMA

Name: Date:

Hosp. No:

D.O.B:

GENERAL COMMENTS (to include comment on weight management and if planning to conceive):

SYMPTOMS:

H/As: Y / N

Triggers:

Type:

Location:

Frequency and Duration:

Severity (1= very mild, 10= very severe):

Number of headaches days per month:

How many of those are severe:

Tinnitus: Y / N : *Whooshing/pulsing* R/L *Pulsatile only* R/L *High Pitched* R/L

Visual Symptoms: Y / N

Diplopia Y / N *TVO* Y / N *Photophobia* Y / N *Phonophobia* Y / N

Comments:

Medication: Y / N

Details (include dosage):

S / E:

DIAGNOSTIC

VA:

Pupils:

Ishihara: R /25 L /25

Weight: kg Height: cm BMI=

Weight loss advice/support given? Y / N / NA

Details:

VF: GVF □ HVF □

OCT: **discs □** **discs and macula □** **macula volume on Heidelberg □**

Completed by: ……………………………

REVIEW

Electrolytes/FBC: Requested in letter to GP □

Not applicable □

Fundus:

Face to face □

Virtual □

Comments:

1. *Symptoms – improved/stable/worse:*
2. *Vision – improved/stable/worse:*
3. *Disc status - improving/stable/deteriorating:*
4. *Weight & BMI – improved/stable/worse and support given (yes/no):*

Actions:

1. *Weight management:*
2. *Medication:*
3. *Review – virtual or face-to-face:*

Reviewed by: …………………….. **Date of review: ……………………..**
