# Supplementary material for: A Quality Assurance Audit of an Orthoptic-Led Virtual Neuro-Ophthalmology Clinic
Source: Br Ir Orthopt J. 2023 Mar 10;19(1):7–14. doi: 10.22599/bioj.289 (PMC10000316; doi:10.22599/bioj.289)
Supplement: Appendix A-2. — General and Tumour Monitorting Proforma. [file bioj-19-1-289-s2.pdf]

GENERAL AND TUMOUR MONITORTING PROFORMA

Name: ________________________________

Date:______________________

Number: ________________________________

**Diagnosis:**		
**Referred by:**		
**Symptoms:**	**For FU patients: BETTER / WORSE / SAME**	
**General Health + Medication**		
**Previous ocular history**		
**Other services:**	Speciality:<br><br>Where?<br><br>Consultant?<br><br>Date last seen?<br><br>Date of last scan (if applicable)?<br><br>Outcome:	
**Social History**	Smoker:	Occupation:
	Alcohol:	Driver:

ASSESSMENT

TEST	RE	LE	Completed by:
**VA**<br>**(c̄ / s̄ gls/PH)**			
**CV**			
**CS**			
**PUPILS**			
**VF**	**Type:** HVF 24-2/HVF 30-2/GVF<br>**Comments:**		
**OCT (DILATED)**	**Type**: DISC/MACULA/BOTH (If VA reduced, please scan Macula)<br>**Comments:**		

REVIEW

	**Comments if unstable:**	
**VA**<br>**STABLE / UNSTABLE**		
**CV**<br>**STABLE / UNSTABLE**		
**PUPILS**<br>**STABLE / UNSTABLE**		
**FIELDS**<br>**STABLE / UNSTABLE**	**RE**	**LE**
**FUNDUS/OCT**<br>**STABLE / UNSTABLE**	**RE**	**LE**
**COMMENTS**		
**FOLLOW UP**	**CLINIC:**	
	**TIMESCALE:**	
**REVIEW**	**COMPLETED BY:**	
	**DATE OF REVIEW:**	
