# Supplementary material for: A Quality Assurance Audit of an Orthoptic-Led Virtual Neuro-Ophthalmology Clinic
Source: Br Ir Orthopt J. 2023 Mar 10;19(1):7–14. doi: 10.22599/bioj.289 (PMC10000316; doi:10.22599/bioj.289)
Supplement: Appendix A-3. — Swollen Discs Clinic – Questionnaire. [file bioj-19-1-289-s3.pdf]

SWOLLEN DISCS CLINIC – Questionnaire

Name: ________________________________

Date:______________________

Number: _______________________________

1. If you have been referred by an optician, was it the first visit to **this** optician? YES / NO

2. Do you suffer from any of these symptoms?

Headache : YES : (please answer the following) NO

- How bad is the headache from 1 to 10: 1 2 3 4 5 6 7 8 9 10
- Does it wake you up from sleeping: YES / NO
- Is it worse in the morning: YES / NO
- Is it worse when coughing, sneezing, or exertion YES / NO
- Is there neck and shoulder pain: YES / NO
- Nature – constant/ pulsatile
- Location- all round head/ behind eyes/ back of the head/ one sided
- Duration-

Transient Visual Obscuration's (temporary loss of vision) YES / NO

Diplopia (double vision) YES / NO

Decreased visual acuity (even with glasses) YES / NO

Pulsatile tinnitus ("whooshing noise" in the ears in time with the pulse) YES / NO

Nausea and vomiting YES / NO

Photophobia (dislike of and pain caused by bright light) YES / NO

Refraction status- short sighted (Myopia)/ Longsighted (Hypermetropia)/ no glasses- orthoptist to perform focimeter check to confirm

3. Weight - Height - BMI:

4. Is there any history of recent gaining weight? YES / NO

5. Past Ocular history: --

----

6. Do you have any other general health issue? Any known migraine---
----

----

7. Medication: --

----

8. Allergy : --

SWOLLEN DISC CLINIC – Assessment and Review

**ASSESSMENT**	RIGHT EYE	LEFT EYE	Completed by:
VISUAL ACUITY<br>(aided / unaided)			
CONTRAST SENSITIVITY<br>(Pelli Robson)			
COLOUR VISION<br>(Ishihara only)			
PUPILS<br>(Orthoptist to assess)			
FIELDS			
OCT (Disc)			
Additional Comments<br>(IOP)			

**REVIEW**	RIGHT EYE	LEFT EYE
VISUAL FUNCTION FINDINGS		
FUNDUS/OCT FINDINGS		
Additional Comments		

Telephone Consultation completed: YES/NO If NO why? ____
Letter dictated: YES/NO If NO why?____

**OUTCOME (please tick and indicate timescale)**	**Discharge**	
	**Doctor**	
	**VNOASF**	
	**Refer to another speciality**	

Completed by: ___________________________________ Date: ____________________________
